# Supplementary material for: Sustainable Biomass Lignin-Based Hydrogels: A Review on Properties, Formulation, and Biomedical Applications
Source: Int J Mol Sci. 2023 Aug 30;24(17):13493. doi: 10.3390/ijms241713493 (PMC10487582; doi:10.3390/ijms241713493)
Supplement: Supplementary file 1 [file ijms-24-13493-s001.zip › ijms-2537513-supplementary.pdf]

International Journal of *Molecular Sciences*

MDPI

*Supplementary Materials: Examples of synthesis methods for lignin-based hydrogels*

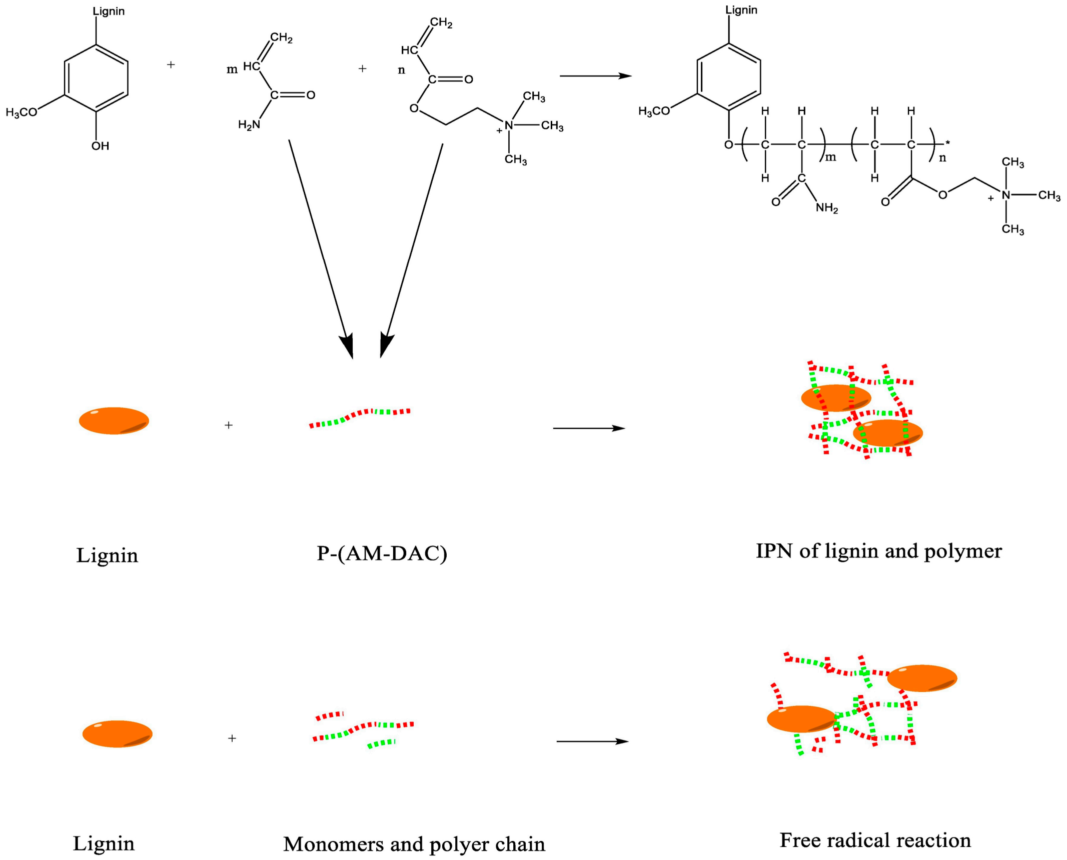


**Figure S1.** Interpenetrating polymer network (IPN) of lignin and crosslinking monomer. This method involves firstly copolymer formation from the monomers, where lignin reacts with the copolymer to form grafted polymers by radical reaction. Secondly, the lignin-grafted polymers penetrated into the network that was formed from copolymer, and thus the IPN network was formed (Reprinted from Meng *et al.*, International Journal of Biological Macromolecules 2019, 135, 1006-1019, doi:10.1016/j.ijbiomac.2019.05.198, with permission from Elsevier. Published under license, Copyright© 2019 Elsevier Ltd.).

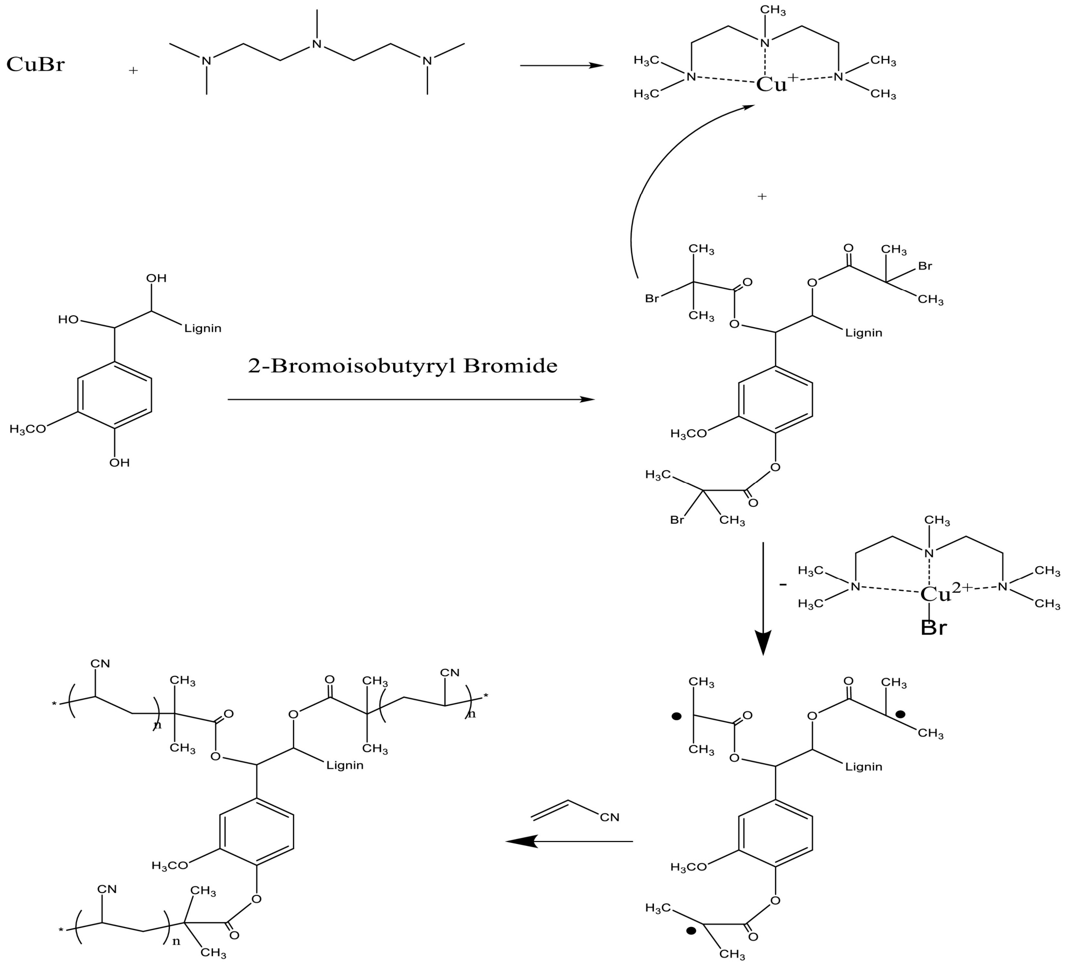


**Figure S2.** An atom transfer radical polymerization (ATRP) process. In order to implement the radical reaction of lignin with the acrylonitrile monomer, the lignin-based organic halide was synthesized as an initiator. Thus, in the ATRP, the lignin-based organic halide functioned as both the initiator and the polymer backbone (Reprinted from Meng *et al.*, International Journal of Biological Macromolecules 2019, 135, 1006-1019, doi:10.1016/j.ijbiomac.2019.05.198, with permission from Elsevier. Published under license, Copyright© 2019 Elsevier Ltd.).

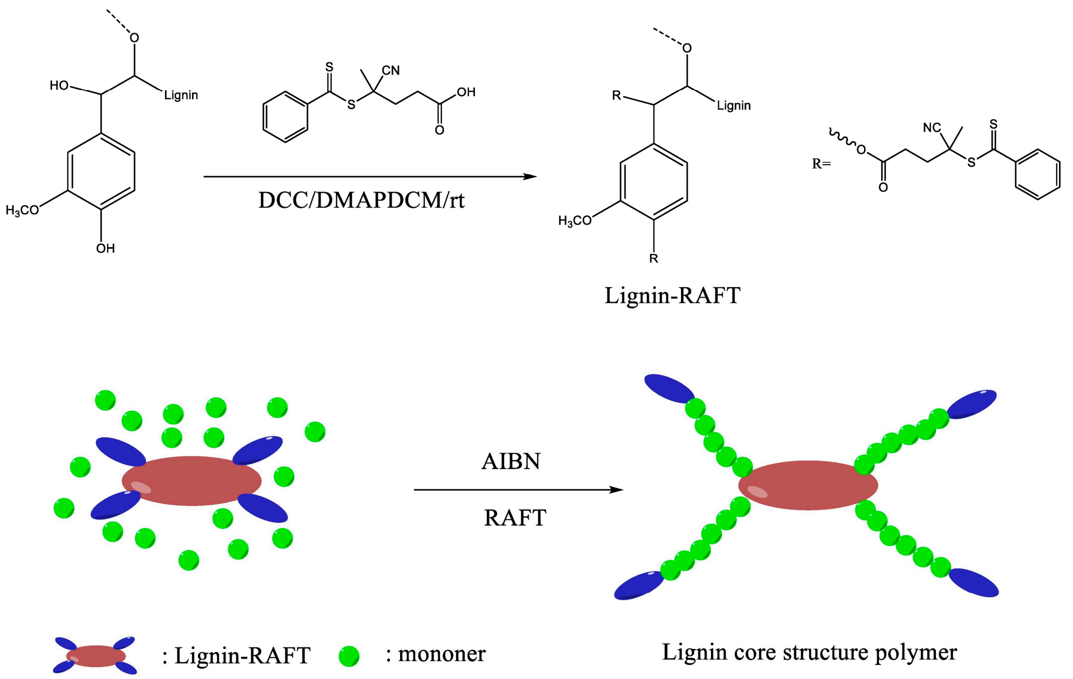


**Figure S3.** A reversible addition-fragmentation transfer (RAFT) process. The lignin based on RAFT is prepared and served as a reaction core first, which was followed by the polymerization of the monomer onto the lignin core through azobisisobutyronitrile (AIBN) initiation and radical reaction (Reprinted from Meng *et al.*, International Journal of Biological Macromolecules 2019, 135, 1006-1019, doi:10.1016/j.ijbiomac.2019.05.198, with permission from Elsevier. Published under license, Copyright© 2019 Elsevier Ltd.).

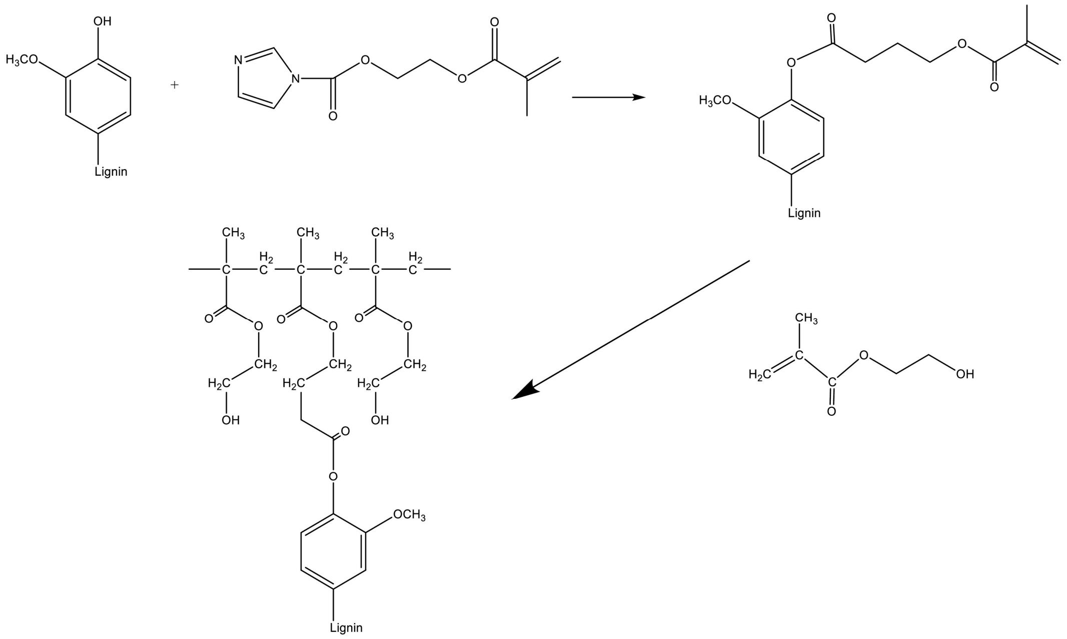


**Figure S4.** Copolymerization of grafted lignin and crosslinking monomer. This method involves introducing a double bond into the lignin structure through an unsaturated monomer, which could render further copolymerization possible. Based on a radical reaction, the unsaturated block is introduced into the lignin backbone through esterification of the phenolic hydroxyl group of the lignin to form an unsaturated grafted lignin (Reprinted from Meng *et al.*, International Journal of Biological Macromolecules 2019, 135, 1006-1019, doi:10.1016/j.ijbiomac.2019.05.198, with permission from Elsevier. Published under license, Copyright© 2019 Elsevier Ltd.).
